# Expositionen mit Fruchtpflanzen in Deutschland im Zeitraum 2010–2019: Auswertung der Datenbank des Gemeinsamen Giftinformationszentrums Erfurt (GGIZ)

**DOI:** 10.1007/s00103-023-03780-7

**Published:** 2023-10-12

**Authors:** Sebastian Wendt, Dagmar Prasa, Christoph Lübbert, Kathrin Begemann, Heike Franke

**Affiliations:** 1 Bereich Infektiologie und Tropenmedizin, Klinik und Poliklinik für Hämatologie, Zelltherapie, Hämostaseologie und Infektiologie, Universitätsklinikum Leipzig, Leipzig, Deutschland; 2 Interdisziplinäres Zentrum für Infektionsmedizin (ZINF), Universitätsklinikum Leipzig, Leipzig, Deutschland; 3 Stabsstelle Krankenhaushygiene, Universitätsklinikum Halle (Saale), Magdeburger Str. 24, 06112 Halle (Saale), Deutschland; 4 Gemeinsames Giftinformationszentrum der Länder Mecklenburg-Vorpommern, Sachsen, Sachsen-Anhalt, Thüringen c/o HELIOS Klinikum Erfurt, Erfurt, Deutschland; 5 Klinik für Infektiologie und Tropenmedizin, Klinikum St. Georg gGmbH, Leipzig, Deutschland; 6 Abteilung Exposition, Bundesinstitut für Risikobewertung (BfR), Berlin, Deutschland; 7 Rudolf-Boehm-Institut für Pharmakologie und Toxikologie, Medizinische Fakultät, Universität Leipzig, Leipzig, Deutschland

**Keywords:** Giftpflanzen, Beeren, Früchte, Epidemiologie, Risikobewertung, Toxikologie, Poisonous plants, Berries, Fruits, Epidemiology, Risk assessment, Toxicology

## Abstract

**Hintergrund:**

Anfragen zu Fruchtpflanzen sind ein häufiger Konsultationsgrund von Giftinformationszentren, wobei hervorzuheben ist, dass es keine großen systematischen Studien zur Giftigkeit auf Grundlage von Expositionsdaten gibt. Ziel der Arbeit ist die Bestimmung des Vergiftungsrisikos durch Fruchtpflanzen in Deutschland.

**Methoden:**

Retrospektive Untersuchung der Daten des Gemeinsamen Giftinformationszentrums Erfurt zu Vergiftungsanfragen bzgl. Fruchtpflanzen (2010–2019) mit ausführlicher Darstellung vorab publizierter Zwischenergebnisse, tabellarischer Handreichung und Pflanzenfotografien als Identifizierungshilfe sowie Trendanalysen.

**Ergebnisse:**

Aus 16.088 Pflanzenexpositionen mit 16.700 Pflanzen wurden 214 verschiedene Fruchtpflanzenarten identifiziert. 45 Fruchtpflanzenarten (21 %) stellten sich als relevant (≥ 30 Anfragen) heraus, davon 6 (2,8 %) als hochrelevant (≥ 300 Anfragen). Allen relevanten Pflanzen wurde eine definierte Risikokategorie (RK) zugeordnet: RK 0 (2; 4,4 %), RK 1 (26; 57,8 %), RK 2 (12; 26,7 %) und RK 3 (5; 11,1 %). Von den Anfragen bezogen sich 6 % (459/7607) auf RK 0, 47,9 % (3645/7607) auf RK 1, 39,3 % auf RK 2 (2986/7607) und 6,8 % (517/7607) auf RK 3. 69,5 % (5284/7607) der Anfragen betrafen Kleinkinder (1 bis < 6 Jahre). Die Expositionsfolgen waren für alle Altersklassen zu 82 % symptomlos, 14,7 % leichtgradig, 3 % mittelschwer und 0,3 % schwer, wobei schwere Vergiftungen durch 7 Pflanzenarten verursacht wurden. Eine Intervention wurde in 66,8 % (5079) der Anfragen eingeleitet. Anfragen betrafen am häufigsten: *Taxus baccata, Ligustrum vulgare, Physalis alkekengi, Prunus laurocerasus, Convallaria majalis, Mahonia* spec., *Sambucus* spec., *Lonicera* spec., *Sorbus aucuparia, Thuja* spec., *Hedera helix* und *Cotoneaster* spec.

**Diskussion:**

Vergiftungen durch Fruchtpflanzen in Deutschland sind selten. Es besteht allerdings ein hoher Informations- und Aufklärungsbedarf.

**Zusatzmaterial online:**

Zusätzliche Informationen sind in der Online-Version dieses Artikels (10.1007/s00103-023-03780-7) enthalten.

## Abstract (en)

**Background:**

Inquiries about fruit plants are a frequent reason for consultation with poison information centers, although it should be emphasized that there are no large systematic studies on toxicity based on exposure data. The aim of this work is to determine the risk of poisoning by fruit plants in Germany.

**Methods:**

Retrospective study of data from the Erfurt Joint Poison Information Center on poisoning inquiries regarding fruit plants (2010–2019) with a detailed presentation of interim results, a tabular handout, plant photos as identification aids, and trend analyses.

**Results:**

From 16,088 plant exposures with 16,700 plants, 214 different fruit plant species were identified. Forty-five fruit plant species (21%) turned out to be relevant (≥ 30 inquiries) and of these, 6 (2.8%) turned out to be highly relevant (≥ 300 inquiries). All relevant plants were assigned a defined risk category (RC): RC 0 (2; 4.4%), RC 1 (26; 57.8%), RC 2 (12; 26.7%), and RC 3 (5; 11.1%). Regarding the inquiries, 6% (459/7607) were related to RC 0; 47.9% (3645/7607) to RC 1; 39.3% to RC 2 (2986/7607); and 6.8% (517/7607) to RC 3. Of the inquiries, 69.5% (5284/7607) were related to young children (1 to < 6 years). Exposure outcomes for all age groups were asymptomatic in 82%, mild in 14.7%, moderate in 3%, and severe in 0.3%, with severe poisoning caused by seven plant species. Interventions were initiated in 66.8% (5079) of the inquiries. Inquiries were most frequently related to *Taxus baccata*, *Ligustrum vulgare*, *Physalis alkekengi*, *Prunus laurocerasus*, *Convallaria majalis*, *Mahonia* spec., *Sambucus* spec., *Lonicera* spec., *Sorbus aucuparia*, *Thuja* spec., *Hedera helix*, and *Cotoneaster* spec.

**Discussion:**

Poisoning by fruit plants in Germany is rare. However, there is a great need for information and education.

## Einleitung

Das Gemeinsame Giftinformationszentrum Erfurt (GGIZ) ist seit 1994 als sogenannter Giftnotruf (Tel. 0361 – 730 730) für die Beratung der Bevölkerung und von Institutionen in den Bundesländern Sachsen, Sachsen-Anhalt, Thüringen und Mecklenburg-Vorpommern verantwortlich. Darüber hinaus existiert seit 2004 auch eine Nachtdienstkooperation mit dem Giftinformationszentrum-Nord in Göttingen und seit 2014 mit der Vergiftungs-Informations-Zentrale in Freiburg. Pro Jahr werden ca. 27.500 Anfragen vom GGIZ bearbeitet, was mehr als einer Verfünffachung seit dem Gründungsjahr 1994 entspricht [2]. Bei jeder Anfrage werden Informationen zum Vergiftungsfall erfasst, z. B. die Charakterisierung des Agens, Datum, Bundesland, Altersgruppe, Symptome, Vergiftungsschwere, Vergiftungsgrund/-motivation und Behandlungsbedarf (jede ärztliche oder nichtärztliche Maßnahme, um Symptome zu lindern). Ein Teil dieser Daten steht auch der interessierten Öffentlichkeit zur Verfügung [3].

Die deutschen Giftinformationszentren (GIZ) und das Bundesinstitut für Risikobewertung (BfR) setzen sich im Sinne des gesundheitlichen Verbraucherschutzes für eine Risikofrüherkennung und -bewertung sowie für ein umfassendes nationales Monitoring von Vergiftungen ein [4, 5]. In der „Kommission zur Bewertung von Vergiftungen“ beim BfR befasst sich ein spezieller Ausschuss mit der Giftigkeit von Pflanzen. Dieser hat 2019 und 2021 eine Risikoneubewertung publiziert [6, 8].

Neben Anfragen zu Haushaltschemikalien und Medikamenten werden in den GIZ häufig auch Pflanzenexpositionen bei Kindern und Jugendlichen (15 %) registriert [1, 2, 6, 7], weshalb Pflanzen im toxikologischen Beratungssetting eine wichtige Bedeutung haben (vgl. Pilzexpositionen: 1–2 %). So werden im Expositionsfall häufig Früchte von Kindern oral aufgenommen [1, 6, 7]. Diese Pflanzenteile sind visuell attraktiv, ähneln oft den Früchten der Nutz- und Nahrungspflanzen und sind häufig ubiquitär zu finden (z. B. auf Sport- und Spielplätzen, in Gartenanlagen oder Stadtparks) und damit gut erreichbar [1]. Aus diesem Grund gibt es eine amtliche Liste giftiger Pflanzen, die nicht an Plätzen angepflanzt werden sollten, die Kindern als Aufenthalts- und Spielort dienen [8]. Toxische Inhaltsstoffe finden sich bei vielen Pflanzen insbesondere auch in den Früchten [9, 10]. Daher ist das Vergiftungsrisiko von Pflanzenarten bei einer Kombination von Giftigkeit der Fruchtkörper, Zugänglichkeit und Attraktivität der Früchte meist als hoch zu bewerten.

Mittels einer Risikoevaluation für Fruchtpflanzen in Deutschland führt die vorliegende Studie die Analysen älterer Auswertungen [6, 7] mit einem anderen Studiendesign und einem aktuelleren Datensatz des GGIZ weiter und leitet daraus Präventionsmaßnahmen und Trends ab.

Zwischenergebnisse der Studie wurden vorab als Kurzmitteilung publiziert [1]. Hier erfolgt die ausführliche Darstellung der kompletten Studie nach einem Peer-Review-Prozess sowie die Veröffentlichung methodischer Einzelheiten inklusive Trendanalysen. Die tabellarische Übersicht aller einbezogenen Fruchtpflanzenarten soll als Handreichung zur Risikoeinschätzung dienen. Zusätzlich werden ausgewählte Pflanzenarten fotografisch präsentiert, um die Identifizierung im Vergiftungsfall zu unterstützen.

## Methoden

Zur Bestimmung des realen Vergiftungsrisikos (Risikobewertung, *Risk Assessment*) wurde eine integrative Analyse von Expositionsdaten für ausgewählte Fruchtpflanzen mit potenziell toxischen Inhaltsstoffen durchgeführt [1]. Grundlage der Risikobewertung ist eine Analyse der Protokolldaten des GGIZ Erfurt für die zugehörigen Bundesländer Mecklenburg-Vorpommern, Sachsen, Sachsen-Anhalt und Thüringen sowie 5 weitere Bundesländer (Bremen, Hamburg, Niedersachsen, Schleswig-Holstein, Baden-Württemberg) im Rahmen der Nachtdienstkooperation für den Zeitraum 2010–2019 [1]. Das Jahr 2020 wurde nicht in den Auswertungszeitraum einbezogen, um mögliche Verzerrungen durch die Coronapandemie (z. B. durch Ausgangsbeschränkungen, kurzfristige Änderung von Freizeitaktivitäten) auszuschließen – ab 2020 kam es am GGIZ Erfurt zu einer verminderten Anfragefrequenz bezüglich Pflanzen, die sich erst jetzt wieder zu „normalisieren“ scheint (mündliche Mitteilung GGIZ Erfurt).

Die Identifizierung der Pflanzen erfolgte entweder durch den Betroffenen bzw. den Pflanzenbesitzer unter Beteiligung des GGIZ oder durch Gärtner, Apotheker oder anderes geschultes Personal. Es wurden nur Expositionen eingeschlossen, die nach Einschätzung des GGIZ mit großer Wahrscheinlichkeit spezifischen Pflanzenarten zuzuordnen waren, d. h., Fälle mit unsicherer Zuordnung zu einer Pflanze (Verdachtsfälle) wurden ausgeschlossen [1]. Eine systematische Unterscheidung zwischen beabsichtigten und unbeabsichtigten Expositionen erfolgte nicht. Der Poisoning Severity Score (PSS) ist die etablierte Grundlage zur Beurteilung der Symptomschwere am GGIZ (s. Onlinematerial; [11]). Die Auswertung der Rohdaten erfolgte mittels MS Excel (Version 16.0, Redmond, USA) und IBM SPSS^®^ (Version 24.0, Armonk, USA).

„Fruchtpflanzen“ werden in der vorliegenden Analyse als breiter botanischer Oberbegriff definiert, der neben Pflanzen mit echten Beeren (Beeren im engeren Sinne mit fleischig-saftiger Fruchtwand und Mehrsamigkeit) auch Pflanzen mit anderen Fruchttypen wie Schoten, Hülsenfrüchten, Kapselfrüchten, Nüssen, Sammelstein‑, Stein- und Apfelfrüchten sowie Zapfen umfasst [12]. Dabei wurden auch Pflanzen einbezogen, die nur sporadisch fruchtartige Strukturen ausbilden (z. B. Engelstrompete). In die Analysen wurden auch Fälle bzw. Expositionen eingeschlossen, an denen u. U. weitere (nicht pflanzliche) Noxen, wie z. B. Medikamente oder Alkohol, beteiligt waren. Die Auswertungen zur Vergiftungsschwere (ohne festgelegte Nachverfolgung) beziehen sich dagegen alleine auf Einzelexpositionen, damit eine Zuordnung zu einer konkreten Pflanzenart möglich ist. Trivialobst und -gemüse (z. B. Gartenkirsche, Tomate), Zimmerpflanzen, Gewürz- und Nutzpflanzen, pflanzliche Zubereitungen und nicht heimische Pflanzen wurden nur in begründeten Fällen in der Auswertung berücksichtigt (u. a. hohe Anfragefrequenz an das GGIZ, populäre Pflanzenarten, „etabliertes“ Suizidmittel, wie z. B. Eibensud). Tierexpositionen wurden gänzlich ausgeschlossen. Ebenfalls ausgeschlossen waren Expositionen mit Stoffen, die nicht natürlicherweise zu diesen Pflanzen gehören, z. B. Biozide, Dünger bzw. andere Chemikalien sowie Infektionserreger (z. B. Fuchsbandwurmeier).

Die Risikokategorien RK 0–3 fußen auf der o. g. Neubewertung von Giftpflanzen durch den Ausschuss „Giftigkeit von Pflanzen“ beim BfR und lehnen sich an die Risikobewertung von Chemikalien an [6]: *RK 0* = ungiftig (keine Symptome), *RK 1* = leicht giftig (allenfalls leichte Symptome), *RK 2* = mittelstark giftig (mittelschwere Symptome), *RK 3* = sehr giftig (schwere Symptome; [1]).

Demnach werden z. B. Fruchtpflanzen der RK 3 zugeordnet, wenn in der Vergangenheit schwere Vergiftungen nach akzidenteller Aufnahme kleiner Mengen berichtet wurden oder wenn hochgiftige Inhaltstoffe in so hohen Konzentrationen in der Pflanze enthalten sein können, dass schwere Vergiftungen bei Kleinkindern bei der Aufnahme kleiner Mengen möglich erscheinen. Die Risikokategorisierung von Hermanns-Clausen et al. ist auch eine Handreichung für Bepflanzungsplanungen im Umfeld von Kindern [6].

In der vorliegenden Arbeit konnten durch die Berechnung eines modifizierten Litovitz-Risikofaktors Fruchtpflanzen mit hohem Vergiftungsrisiko identifiziert werden [1, 7]: Dabei wurden die schweren und mittelschweren Intoxikationen addiert und auf 100 Gesamtexpositionen bezogen. Die Definition für „relevante Fruchtpflanzen“ wurde auf eine Anfragehäufigkeit von mindestens 30 pro Einzelpflanze im Studienzeitraum festgelegt, „hochrelevante Fruchtpflanzen“ schließen dagegen mindestens 300 Anfragen pro Einzelpflanze im Analysezeitraum ein [1].

Anfragetrends im Zeitraum 2010–2019 wurden mittels Mann-Kendall-𝜏-Tendenztests analysiert.

## Ergebnisse

### Expositionsbetrachtungen

Für den Abfragezeitraum 2010–2019 konnten von insgesamt 174.969 Expositionen am GGIZ 16.088 einer Pflanzenexposition zugeordnet werden. Da an einer Exposition aber auch mehrere Pflanzen beteiligt sein können, beträgt die Gesamtanzahl aller Expositionspflanzen 16.700 (davon 612 Pflanzenexpositionen mit > 1 Pflanzenart). Davon gehören insgesamt 8967 (53,7 %) zu Fruchtpflanzen nach der o. g. Definition, wovon sich 7607 (84,8 %) Anfragen auf relevante Fruchtpflanzen (≥ 30 Anfragen pro Analysezeitraum) beziehen [1]. 747 verschiedene Pflanzenarten bzw. -gattungen waren mit Anfragen dokumentiert, davon 214 Fruchtpflanzenarten (28,7 %), wovon 45 (21 %) das Kriterium der Relevanz erfüllten (≥ 30 Anfragen pro Analysezeitraum; [1]). Von diesen 45 relevanten Fruchtpflanzen gehörten 2 (4,4 %) zu RK 0 und 26 (57,8 %) zu RK 1 sowie 12 (26,7 %) zu RK 2 und 5 (11,1 %) zu RK 3. Wie in der Vorabpublikation beschrieben, bezogen sich von insgesamt 7607 Einzelanfragen zu relevanten Fruchtpflanzen 459 (6 %) Anfragen auf RK 0 und 3645 (47,9 %) auf RK 1 sowie 2986 (39,3 %) auf RK 2 und 517 (6,8 %) auf RK 3 [1]**.**

Mit 5284 (69,5 %) Einzelexpositionen bilden Kleinkinder (1 bis < 6 Jahre) die häufigste Expositionsgruppe; es folgen Volljährige (18 bis < 65 Jahre, Senioren ab 65 Jahren, Erwachsene mit unbekanntem Alter) mit 1091 Expositionen (14,3 %), Schulkinder (6 bis < 14 Jahre) mit 727 (9,6 %) Expositionen, Babys (bis 1 Jahr) mit 265 (3,5 %) und Jugendliche (14 bis < 18 Jahre) mit 83 Fällen (1,1 %; [1]). An 59 Anfragen (0,78 %) waren mehrere Personen unterschiedlicher Altersgruppen beteiligt.

Patienten mit Exposition zu relevanten Fruchtpflanzen als Hauptnoxe (*n* = 7186) blieben in 82 % (5891) aller Fälle symptomfrei, bei 14,7 % (1059) zeigten sich nur leichte Symptome, bei 3 % (212) mittelschwere und bei 0,3 % (24) wurde die Symptomatik als „schwer“ eingeschätzt [1]. Unter den schweren Symptomen fanden sich 5 Todesfälle (0,07 %), bei denen es sich um Suizide handelte [1]. Bei 5,9 % (421) der Exponierten waren die Symptome entweder unbekannt oder nicht einschätzbar (Tab. 1).

**Table 1:** 

Pflanze	Trivialname	RK	Giftige Pflanzenteile	Relevante Inhaltsstoffe (Auswahl)	Anfragen 2010–2019	Symptomlos	Leicht	Mäßig	Schwer/verstorben	Nicht einschätzbar	Unbekannt	Mod. Litovitz-Faktor
*Aesculus *spec.	Rosskastanie	1	Samen	Aescin, Aescigenin	**113**	91	13	1	0	5	3	**0,88**
*Amelanchier *spec.	Felsenbirne	1	Blätter, Samen	Cyanogene Glykoside	**31**	24	7	0	0	0	0	**0**
*Aronia *spec.	Apfelbeere, Zwergvogelbeere	0	Beeren, inkl. Samen	Amygdalin	**31**	21	8	0	0	2	0	**0**
*Arum *spec.	Aronstab	2	Alle, besonders Früchte	Aroin, Aronin, Nicotin	**186**	84	82	6	0	10	4	**3,23**
*Atropa belladonna*	Tollkirsche	3	Alle	Atropin, Scopolamin, L‑Hyoscyamin	**43**	16	12	9	3	2	1	**27,91**
*Berberis* spec.	Berberitze, Sauerdorn	1	Wurzel, Stammrinde (Früchte enthalten nur Spuren von Berberin)	Berberin	**45**	37	6	0	0	2	0	**0**
*Brugmansia *spec.	Engelstrompete	3	Alle, besonders Blätter, Blüten, Samen	Scopolamin, L‑Hyoscyamin, Atropin	**114**	27	41	28	3	10	5	**27,19**
*Caragana arborescens*	Gemeiner Erbsenstrauch	1	Alle	Cytisin	**43**	37	5	0	0	0	1	**0**
*Colchicum autumnale*	Herbstzeitlose	3	Alle, besonders Wurzelknolle, Blüten, Samen, Blätter	Colchicin	**207**	89	74	25	4	14	1	**14,01**
*Convallaria majalis*	Maiglöckchen	2	Blüten, Samen, Blätter	Convallatoxin	**418**	328	47	7	0	25	11	**1,67**
*Cotoneaster *spec.	Zwergmispel	1	Rinde, Blätter, Blüten, Früchte	Prunasin, Amygdalin	**224**	200	15	5	0	1	3	**2,23**
*Daphne mezereum*	Echter Seidelbast	2	Alle, besonders Rinde und rote Beeren	Daphnetoxin	**37**	27	7	0	0	2	1	**0**
*Datura *spec.	Stechapfel	3	Alle, besonders Blätter, Blüten, Samen	L‑Hyoscyamin, Atropin, Scopolamin	**74**	18	31	17	1	5	2	**24,32**
*Euonymus *spec.	Pfaffenhütchen	1	Alle, besonders Früchte	Evonosid	**58**	46	9	0	0	1	2	**0**
*Fagus *spec.	Buche	1	Früchte	Saponine	**64**	32	29	0	0	3	0	**0**
*Frangula alnus*	Faulbaum	1	Alle	Glucofrangulin A/B, Frangulin A/B	**30**	29	0	0	0	0	1	**0**
*Hedera helix*	Gemeiner Efeu	2	Alle, besonders Früchte, Blätter	Hederine, Falcarinol	**228**	158	41	8	0	17	4	**3,51**
*Ilex aquifolium*	Europäische Stechpalme	1	Früchte, Blätter	Rutin, Ursolsäure	**141**	121	11	3	0	5	1	**2,13**
*Laburnum anagyroides*	Gemeiner Goldregen	2	Alle, besonders die bohnenartigen Hülsen	Cytisin	**108**	75	20	2	0	6	5	**1,85**
*Lathyrus *spec.	Platterbse, Gartenwicke	1	Samen, Früchte	Arbutin	**178**	167	8	0	0	2	1	**0**
*Ligustrum vulgare*	Gewöhnlicher Liguster	1	Beeren, Rinde, Blätter	Ligustrosid, Syringin	**640**	568	51	3	0	11	7	**0,47**
*Lonicera *spec	Heckenkirsche, Geißblatt	1	Früchte	Triterpenoidsaponine	**291**	268	17	2	0	2	2	**0,69**
*Mahonia *spec.	Mahonie	1	Wurzel, Rinde, Blätter, Samen	Berberin, Berbamin	**311**	285	13	4	0	4	5	**1,61**
*Parthenocissus *spec.	Jungfernrebe, Wilder Wein	2	Früchte	Oxalsäure, Calciumoxalat	**105**	84	13	2	0	5	1	**1,90**
*Physalis alkekengi*	Lampionblume, Judenkirsche, Blasenkirsche	0	Blätter, Wurzel, unreife Beeren	Physaline, Solanin	**428**	399	15	2	0	4	8	**0,47**
*Phytolacca *spec.	Kermesbeere	2	Alle, besonders Früchte, Samen	Phytolaccatoxin	**96**	87	8	1	0	0	0	**1,04**
*Polygonatum *spec.	Weißwurz, Salomonsiegel	1	Alle, besonders Beeren	Acetidin-2-carbonsäure, Chelidonsäure	**31**	23	4	0	0	1	3	**0**
*Prunus avium*	Vogelkirsche	1	Samen	Amygdalin, Prunasin	**31**	21	5	2	0	2	1	**6,45**
*Prunus cerasifera*	Kirschpflaume, Myrobalane, Blutpflaume	1	Samen	Amygdalin, Prunasin	**33**	28	3	0	0	2	0	**0**
*Prunus laurocerasus*	Lorbeerkirsche	1	Blätter, Samen, Früchte	Amygdalin, Prunasin	**419**	349	39	3	0	17	11	**0,72**
*Pyracantha *spec.	Feuerdorn	1	Samen	Rutin, Chlorogensäure	**80**	71	8	0	0	1	0	**0**
*Quercus *spec.	Eiche	1	Alle, besonders Früchte, Blätter, Rinde	Ellagitannine, Gallotannine	**133**	115	14	1	0	1	2	**0,75**
*Ricinus communis*	Wunderbaum	3	Samen	Ricin, Ricinin	**79**	28	16	20	3	8	4	**29,11**
*Robinia pseudoacacia*	Gewöhnliche Robinie, Scheinakazie	2	Samen	Robin, Phasin	**116**	86	19	4	0	5	2	**3,45**
*Sambucus *spec.	Holunder	2	Blätter, Früchte	Sambunigrin, Prunasin	**295**	152	110	15	0	12	6	**5,08**
*Solanum dulcamara*	Bittersüßer Nachtschatten	1	Unreife Früchte, Stängel, Wurzel	Tomatidin, Solasodin	**30**	27	1	1	0	0	1	**3,33**
*Solanum nigrum*	Schwarzer Nachtschatten	1	Unreife Früchte, Blätter	Solanin, Solasodin, Solamargin	**62**	52	8	0	0	1	1	**0**
*Solanum pseudocapsicum*	Korallenstrauch, Korallenkirsche	1	Früchte	Solanocapsin	**69**	59	7	0	0	2	1	**0**
*Sorbus aucuparia*	Vogelbeere, Eberesche	1	Samen, rohe Beeren	Parasorbinsäure, Aucuparin	**248**	199	30	8	0	5	6	**3,23**
*Symphoricarpos albus*	Gewöhnliche Schneebeere, Knallerbse	1	Blätter, Wurzel	Saponine	**206**	158	32	0	0	6	10	**0**
*Taxus baccata*	Europäische Eibe	2	Alle außer Samenmantel	Taxine, Taxol, Taxane	**1120**	904	115	19	9	43	30	**2,50**
*Thuja *spec.	Lebensbaum	2	Alle, besonders Blätter	Thujon	**233**	158	45	11	1	17	1	**5,15**
*Viburnum *spec.	Schneeball	1	Rinde, Blätter, Früchte	Viburnin	**65**	61	3	0	0	0	1	**0**
*Viscum album*	Mistel	1	Alle, besonders Früchte	Viscotoxine	**69**	56	7	0	0	3	3	**0**
*Wisteria sinensis*	Chinesischer Blauregen	2	Alle, besonders Samen	Wistarin	**44**	26	10	3	0	3	2	**6,82**

Behandlungsbedarf mit einem Spektrum aus Laienbehandlung oder ärztlicher Behandlung (ambulant oder stationär) oder eine „Kombinationsbehandlung“ bestand bei 5079 Personen (66,8 %). In 2251 Fällen (29,6 %) war keine Therapie nötig, in 277 Fällen (3,6 %) war der Behandlungsbedarf nicht einschätzbar. Bei 710 Fällen (9,3 %) war mindestens eine ambulante Therapie notwendig, bei 643 Fällen (8,5 %) eine stationäre Therapie. 1128 (14,3 %) Expositionen konnten durch Laienbehandlung therapiert werden, bei 1760 (23,1 %) bzw. bei 838 (11 %) Fällen wurde zusätzlich ein Arzt bzw. eine Klinik konsultiert.

3347 (44 %) der 7607 Anfragen zu relevanten Fruchtpflanzen gingen auf Anrufe aus medizinischen Kliniken zurück.

### Auswertung einzelner Fruchtpflanzen

45 von 214 Fruchtpflanzen (21 %) konnten bei einem *Cut-off *von mindestens 30 Anfragen pro Analysezeitraum als relevant eingestuft werden, 6 (2,8 %) als hochrelevant (≥ 300 Anfragen pro Analysezeitraum; [1]).

**Figure 1:**
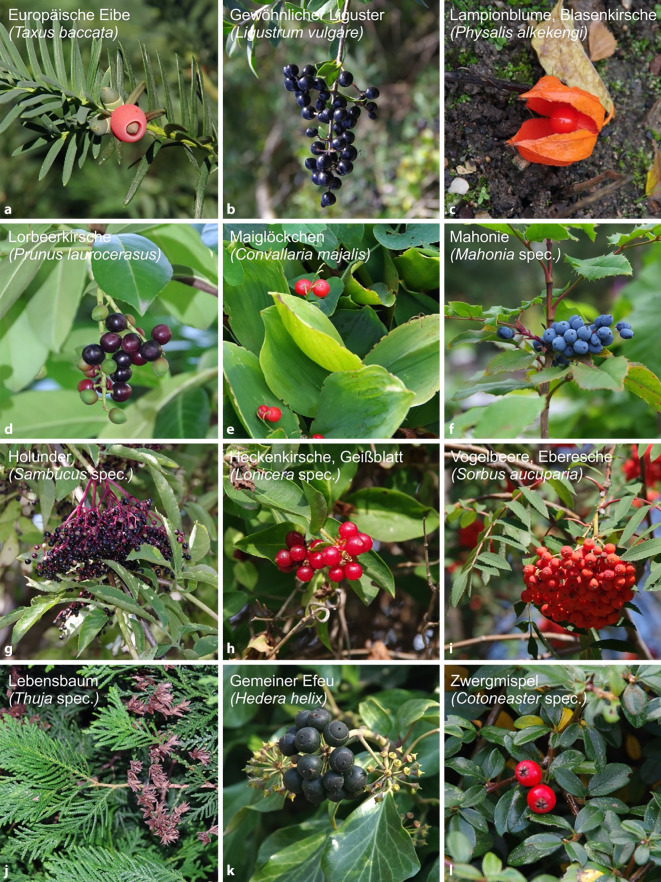

Die 12 am häufigsten angefragten Fruchtpflanzen (insgesamt 4855 Anfragen) in absteigender Häufigkeit sind (Abb. 1 und 2): *Taxus baccata* (Europäische Eibe, *n* = 1120), *Ligustrum vulgare* (Gewöhnlicher Liguster, *n* = 640), *Physalis alkekengi* (Lampionblume/Blasenkirsche, *n* = 428), *Prunus laurocerasus* (Lorbeerkirsche, *n* = 419), *Convallaria majalis* (Maiglöckchen, *n* = 418), *Mahonia *spec. (Mahonie, *n* = 311), *Sambucus *spec. (Holunder, *n* = 295), *Lonicera* spec. (Heckenkirsche/Geißblatt, *n* = 291), *Sorbus aucuparia* (Vogelbeere/Eberesche, *n* = 248), *Thuja* spec. (Lebensbaum, *n* = 233), *Hedera helix* (Gemeiner Efeu, *n* = 228) und *Cotoneaster* spec. (Zwergmispel, *n* = 224).

**Figure 2:**
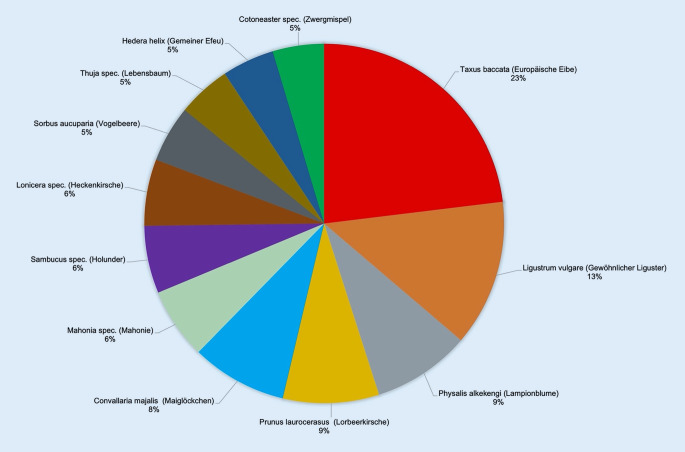

**Figure 3:**
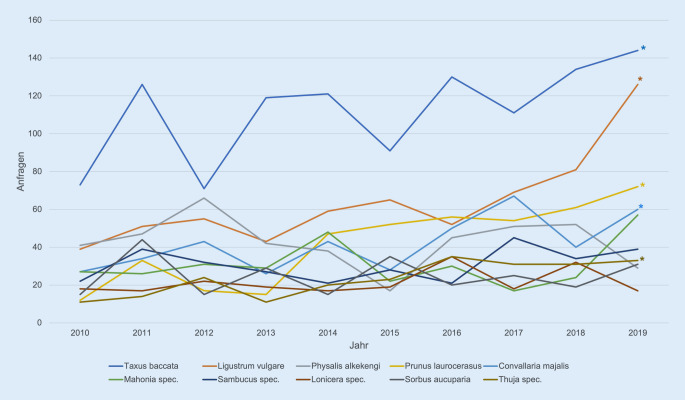

In der Mann-Kendall-𝜏-Tendenzanalyse zeigen sich für *Taxus baccata* (𝜏 = 0,511; *p* = 0,023), *Ligustrum vulgare* (𝜏 = 0,778; *p* < 0,01), *Prunus laurocerasus* (𝜏 = 0,822; *p* < 0,01), *Convallaria majalis* (𝜏 = 0,494; *p* < 0,01) und *Thuja* spec. (𝜏 = 0,659; *p* < 0,01) deutlich ansteigende Anfragetrends innerhalb der letzten 10 Jahre (Abb. 3).

Symptome wurden nach Exposition mit 44 der 45 relevanten Fruchtpflanzen berichtet (Tab. 1). Nur bei Exposition mit *Frangula alnus* (Faulbaum) wurden keine Symptome gemeldet (29 symptomlos, 1 unbekannt). Zu Symptomen kam es am häufigsten bei *Taxus baccata* (*n* = 143), *Sambucus *spec. (*n* = 125), *Colchicum autumnale* (*n* = 103), *Arum* spec. (Aronstab, *n* = 88), *Brugmansia* spec. (Engelstrompete, *n* = 72), *Thuja* spec. (*n* = 57), *Ligustrum vulgare* (*n* = 54), *Convallaria majalis* (*n* = 54), *Datura* spec. (Stechapfel, *n* = 49), *Hedera helix* (*n* = 49) und *Ricinus communis* (Wunderbaum, *n* = 39).

**Figure 4:**
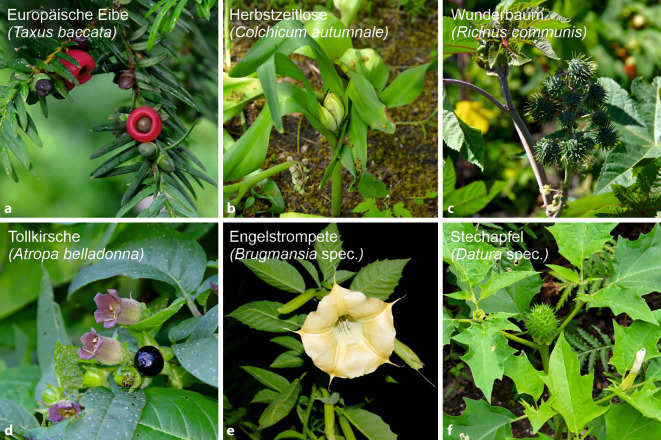

7 Fruchtpflanzen waren als Hauptnoxe für 24 schwere Vergiftungen verantwortlich (absteigende Häufigkeit; [1]): *Taxus baccata* (*n* = 9), *Colchicum autumnale* (*n* = 4), *Ricinus communis* (*n* = 3), *Atropa belladonna* (Tollkirsche, *n* = 3), *Brugmansia* spec. (*n* = 3), *Datura* spec. (*n* = 1; Abb. 4) und *Thuja* spec. (Lebensbaum, *n* = 1; Abb. 1, [1]). Bei den schweren Vergiftungen gab es 5 Todesfälle (Suizide) durch Herbstzeitlose (*n* = 1), Wunderbaum (*n* = 1) und Europäische Eibe (*n* = 3; [1]). Diese Pflanzen sind in der retrospektiven Auswertung zugleich auch am häufigsten mit Suizidversuchen assoziiert gewesen (*n* = 121; [1]). Mittelschwere Vergiftungssymptome kamen bei 212 Expositionen (27 Pflanzenarten) und leichte Symptome bei 1059 Expositionen (43 Pflanzenarten) vor (Tab. 1).

Die höchsten modifizierten Litovitz-Risikofaktoren ergeben sich für folgende Pflanzen: *Ricinus communis* (29), *Atropa belladonna* (28), *Brugmansia* spec. (27), *Datura* spec. (24) und *Colchicum autumnale* (14; Tab. 1**, **Abb. 4, [1]). *Taxus baccata* hat mit 2,5 einen relativ niedrigen Litovitz-Risikofaktor.

## Diskussion

Zur Bestimmung des realen Vergiftungsrisikos wurde eine Analyse von Expositionsdaten für Fruchtpflanzen mit potenziell toxischen Inhaltsstoffen im Einzugsbereich des GGIZ Erfurt für den Zeitraum 2010–2019 durchgeführt (Risikobewertung, *Risk Assessment*; [1]).

In diesem die Coronapandemie nicht umfassenden Analysezeitraum waren Anfragen zu Pflanzenvergiftungen im Kindes- und Jugendalter unter allen Vergiftungsanfragen am dritthäufigsten (15 %). Der Anteil der entsprechenden Anfragen im Erwachsenenalter war mit 2 % vergleichsweise wenig relevant (aktuelle Anfragestatistiken des GGIZ: http://www.ggiz-erfurt.de/giftinformation.html). Das Expositionsrisiko ist im Kindesalter gegenüber bestimmten Noxen erhöht [1]: Kleinkinder erkunden ihre Umgebung noch mit dem Mund. Darüber hinaus sind Kinder oft noch nicht in der Lage, Pflanzen und deren Gefahrenpotenziale korrekt einzuordnen. Auffällig ist, dass die häufigsten Expositionspflanzen in unserer Studie Früchte (53,7 %) hatten, die möglicherweise gerade auf Kinder verlockend wirken [1]. Unsere Ergebnisse bestätigen Aussagen früherer Studien, nach denen Kleinkinder mehrheitlich Früchte (gefolgt von Blättern/Nadeln, Blüten, Wurzeln) ingestieren [7]. Auch in einer älteren retrospektiven Auswertung zu Vergiftungsanfragen im Zeitraum 1998–2004 bei deutschen Giftinformationszentren bilden 9 der 10 am häufigsten angefragten Pflanzenarten auffällige Fruchtstrukturen aus (*Ficus *spec.*, Taxus *spec.*, Prunus *spec.*, Sorbus *spec.*, Ligustrum *spec.*, Lonicera *spec.*, Mahonia* spec.*, Cotoneaster* spec.*, Ilex* spec.; [13]). Dass Kinder im Allgemeinen von Pflanzenvergiftungen häufiger betroffen sind, zeigte sich auch in einer Studie, die vom Ausschuss „Giftigkeit von Pflanzen“ der BfR-Kommission zur Bewertung von Vergiftungen durchgeführt wurde [7].

Konform zum jahreszeitlichen Verlauf ist ab Juli eine deutliche Zunahme der Gesamtanfragen zu Pflanzen zu verzeichnen (unveröffentlichte GGIZ-Daten), die bis Oktober anhält – und damit auch die Reifezeit vieler Früchte widerspiegelt. Akzidentelle Expositionen zu potenziell giftigen Früchten sind zwar häufig, aber selten symptomatisch (82 % asymptomatisch, mod. Litovitz-Faktor für alle Fruchtpflanzen = 4; [1]). Ähnlich stellt sich dies – untersucht im Kollektiv von Kindern im Alter von 0–14 Jahren – auch in einer älteren deutschen Studie von Pietsch et al. dar, wo knapp 90 % der Betroffenen keinerlei Symptome zeigten [13]. Häufig wird von Anrufern ein höheres Vergiftungsrisiko vermutet als tatsächlich vorhanden.

Die „Giftigkeit“ von Fruchtpflanzen ist abhängig von den Inhaltsstoffen, der Ingestionsmenge (Dosis), dem Reifegrad der Früchte, vom Pflanzenalter und vom Wachstumsstandort (Wetter, Klima; [1]). Schätzungsweise 150 der 3000 indigenen Pflanzenarten sind demnach potenziell giftig [9]. Aber auch individuelle Faktoren wie die „Empfindlichkeit“ des Organismus (Enzyme, Alter), der Aufnahmeweg (oral, dermal, inhalativ, okulär, intravasal) und die Expositionsdauer (akut, subakut, chronisch) sind für die Giftwirkung relevant [9]. Bei einigen Früchten wie bei denen von *Taxus baccata, Prunus laurocerasus* und *Daphne mezereum *(Echter Seidelbast) macht es einen Unterschied, ob das attraktive, aber weniger oder sogar ungiftige Perikarp („Fruchtfleisch“) bzw. der Arillus („Samenmantel“) oder der weniger attraktive, aber relativ toxische Samen verzehrt wird. Darüber hinaus ist der Zerkleinerungsgrad (beispielsweise durch Zerkauen) der Samen für die Freisetzung der Inhaltsstoffe und damit für die toxische Wirkung relevant.

Insgesamt 7 Pflanzen führten bei Ingestion zu schweren Symptomen: *Taxus baccata* (RK 2), *Colchicum autumnale* (RK 3), *Ricinus communis* (RK 3), *Brugmansia* spec. (RK 3), *Atropa belladonna* (RK 3), *Datura* spec. (RK 3) und *Thuja* spec. (RK 2; Abb. 1 und 4) – wie zu erwarten, überwiegend Pflanzen der RK 3. Die Anfragezahlen zu Fruchtpflanzen der RK 3 sind allerdings vergleichsweise niedrig. Bereits in früheren Studien wurden *Brugmansia* spec. und *Thuja* spec. als Auslöser für schwere Vergiftungen bei Kindern identifiziert – diese beiden Fruchtpflanzenarten scheinen also schon über längere Zeit von hoher Relevanz zu sein (z. B. [13]).

Wie auch in einer anderen Studie zu 2 GIZ gezeigt wurde [7], wird am häufigsten Rat zu *Taxus baccata* gesucht (*n* = 1120). Für diese Pflanze waren auch die meisten absoluten schweren Fälle zu verzeichnen (*n* = 9, davon 3 Todesfälle) – allerdings bei geringer relativer Symptomrate (91 % symptomlos oder leicht symptomatisch, modifizierter Litovitz-Faktor 2,5). *Taxus baccata* wurde in RK 2 heruntergestuft, da sich – trotz sehr toxischer Inhaltsstoffe – nur ein relativ geringes reales Vergiftungsrisiko bei Kindern im Rahmen von Unfällen und Aufnahme kleiner Mengen ergeben hatte [6]. Dies lässt sich auch mit den Daten dieser Studie untermauern. Das Risiko, das von der Pflanze – außerhalb von Pflanzenzubereitungen (z. B. bei suizidalen Absichten) – ausgeht, ist daher geringer, als man zunächst vermuten könnte. Hervorzuheben ist, dass in 6 schweren bzw. mittelschweren Fällen Sud bzw. „Tee“ von *Taxus baccata* als konzentrierte Zubereitung konsumiert wurde, wobei dann auch von artifiziell hohen Dosen toxischer Wirkstoffe auszugehen ist. Derartige Fälle wurden jedoch in der Risikobewertung nicht berücksichtigt, da es sich einerseits um beabsichtigte Expositionen in selbstschädigender Absicht mit Ingestion größerer Mengen und andererseits um die Aufnahme von Pflanzenzubereitungen handelt [6]. Die hier vorgestellten Ergebnisse zeigen jedoch, dass beabsichtigte Ingestionen (auch von Pflanzenzubereitungen) für die ärztliche Praxis relevant sein können und absichtliche Vergiftungen mit Pflanzenzubereitungen bei Suiziden und Suizidversuchen – zumindest mit *Taxus baccata* – eine Rolle spielen [14]. Allerdings wurden Vergiftungsabsichten in dieser Studie nicht systematisch untersucht, sondern nur in ausgewählten Fällen nacherfasst.

Ein hoher Informations- und Aufklärungsbedarf besteht aufgrund vieler Anfragen bei folgenden 12 Fruchtpflanzen (Abb. 1 und 2, [1]): *Taxus baccata, Ligustrum vulgare, Physalis alkekengi, Prunus laurocerasus, Convallaria majalis, Mahonia *spec., *Sambucus *spec., *Lonicera* spec., *Sorbus aucuparia, Thuja* spec., *Hedera helix* und *Cotoneaster* spec. Allerdings gehört keine dieser Pflanzen RK 3 an. Hier differieren das tatsächliche Vergiftungsrisiko („risk“) und die Höhe des Informationsbedarfs bezüglich dieser Pflanzen. Das liegt vermutlich an geringen botanischen bzw. toxikologischen Kenntnissen der Anrufer.

Die steigende Zahl von Anfragen zu *Taxus baccata, Ligustrum vulgare* und *Prunus laurocerasus* ist auffällig. Es handelt sich hierbei um Pflanzen, die aufgrund ihrer geobotanischen Ökologie mit wärmeren Klimaten gut zurechtkommen und eine zunehmende Verbreitung in Gärten und Parkanlagen haben [15]. Inwiefern der Anfragetrend damit indirekt mit dem Klimawandel im Zusammenhang steht, muss mangels ausreichender Daten vorerst offenbleiben.

Die Hochstufung von *Sambucus *spec. in RK 2 in der Publikation von Hermanns-Clausen et al. aufgrund relativ vieler und symptomatischer Expositionen lässt sich auch durch unsere Befunde unterlegen [6]: Bei relativ hoher Expositionsrate waren gleichzeitig viele Personen symptomatisch (295 Fälle, davon 125 (42,4 %) symptomatisch, modifizierter Litovitz-Faktor: 5,08). *Sambucus *spec. ist in Deutschland weitverbreitet und beliebt, wobei die Früchte erst nach einem Erhitzungsprozess oder durch Alkoholmazeration bekömmlich werden [9]. Das Vergiftungspotenzial dieser Pflanze sollte daher nicht unterschätzt werden. Die Herunterstufung von *Prunus laurocerasus* nach RK 1 kann durch unsere Befunde ebenfalls unterstützt werden: Es gab nur ca. 10 % symptomatische Fälle mit einem modifizierten Litovitz-Faktor von 0,72 – darunter befand sich kein Fall mit schwerer Symptomatik.

Problematisch ist, dass ein Großteil der Expositionen mit Fruchtpflanzen in Deutschland Kleinkinder im Alter von 1–5 Jahren betrifft. Daher sollten edukative Präventionsmaßnahmen so früh wie möglich und nicht erst im schulischen Sachkundeunterricht umgesetzt werden.

Weiterführende Analysen von Expositionsdaten anderer deutscher GIZ, z. B. auch im Hinblick auf regionale Besonderheiten bezüglich der Abundanz und Giftigkeit einzelner Fruchtpflanzen sowie auf ein sich möglicherweise wandelndes „Vergiftungsspektrum“ aufgrund des Klimawandels oder auch zur Aufklärung bestehender Risikodiskrepanzen, sind wünschenswert [16]. Trotz des großen Einzugsgebietes des GGIZ und der Nachtdienstkooperation mit dem Giftinformationszentrum-Nord in Göttingen und seit 2014 mit der Vergiftungs-Informations-Zentrale in Freiburg sind die GGIZ-Daten mit 9 eingeschlossenen Bundesländern möglicherweise nicht repräsentativ genug, um auf die gesamte Bundesrepublik übertragen werden zu können. Es ist denkbar, dass bestimmte Fruchtpflanzenarten in den einbezogenen Bundesländern häufiger oder seltener als in anderen Regionen Deutschlands vorkommen. Es wurde bereits erwähnt, dass die Wirkstoffgehalte von Giftpflanzen, und damit die potenziellen Vergiftungsrisiken, auch von lokalen Faktoren abhängen [9].

Wie bei jedem retrospektiven Studiendesign besteht die Möglichkeit, dass der zugrunde liegende Datensatz nicht vollständig ist. Da die GIZ-Meldungen freiwillig sind, oft durch Laien ohne medizinische oder toxikologische Vorkenntnisse übermittelt werden und wahrscheinlich Anrufe eher bei symptomatischen Fällen stattfinden, könnten die Daten auch einer gewissen Verzerrung (*Reporting Bias*) unterliegen. Bei den „symptomatischen“ Patienten mit angenommenem kausalen Zusammenhang zu einer bestimmten Pflanzenart kann möglicherweise auch der Noceboeffekt (negative gesundheitliche Auswirkung einer wahrgenommenen Exposition ohne echten kausalen Zusammenhang) eine Rolle gespielt haben, der evtl. zu einer Überbewertung des Risikos geführt hat [7, 17]. Die Nachverfolgungs- und Verifikationsmöglichkeiten waren im Rahmen der Telefongespräche begrenzt [1]. So beschreibt die Symptomschwere den maximalen Schweregrad ohne definierte Nachverfolgung. Auch war nicht immer klar dokumentiert, um welchen Pflanzenteil es sich im Einzelfall genau gehandelt hatte. Die Studie erlaubt außerdem keine Aussage über die Giftigkeit von Zubereitungen von Fruchtpflanzen zu medizinischen oder psychoaktiven Zwecken; diese kann aufgrund anderer Wirkstoffkonzentrationen bzw. -zusammensetzungen abweichen und bedarf einer separaten Auswertung.

Letztendlich sollte ein kontinuierliches, tagesaktuelles, nationales Monitoring- bzw. Toxikovigilanzsystem mit jährlicher Berichterstattung für Vergiftungsfälle geschaffen werden, das auf standardisierten und aggregierten Daten aller GIZ, des BfR und weiterer Akteure fußt. Dieses würde die Risikoeinschätzung (*Risk Assessment*) auch bei Fruchtpflanzen erleichtern, regionale Besonderheiten identifizieren können und ein frühestmögliches Eingreifen, beispielsweise beim „Ausbruchsgeschehen“ im Sinne jahreszeitlicher Häufungen, ermöglichen. Andere Länder wie die USA, die Niederlande, die Schweiz und Schweden verfügen bereits über derartige Systeme [18].

## Fazit

Für eine realistische Einschätzung und Etablierung von Schutzmaßnahmen bezüglich Fruchtpflanzenvergiftungen muss das tatsächliche Risiko unter Berücksichtigung von Expositionsdaten beurteilt werden (Risikobewertung, *Risk Assessment*). Die reine Betrachtung von pflanzlichen Inhaltsstoffen und deren toxikologischem Potenzial ist für die Risikobewertung nicht ausreichend [1].

Durch unsere Studie konnten 45 Fruchtpflanzen mit hoher bzw. 6 mit höchster Relevanz für die deutsche Bevölkerung identifiziert werden [1]. Eine besondere Schutzgruppe stellen in diesem Zusammenhang Kinder im Alter von 1–5 Jahren dar [1].

Zwar sind lebensbedrohliche Vergiftungen durch Fruchtpflanzen in Deutschland insgesamt sehr selten; die hohen Anruf- und Nachfragefrequenzen am GGIZ lassen aber auf fehlende botanische bzw. toxikologische Kenntnisse in der Bevölkerung schließen, denen mit entsprechenden Informationsangeboten entgegengewirkt werden sollte [1].

### Supplementary Information




